# Supplementary material for: Association between oral microbiome and seven types of cancers in East Asian population: a two-sample Mendelian randomization analysis
Source: Front Mol Biosci. 2023 Nov 21;10:1327893. doi: 10.3389/fmolb.2023.1327893 (PMC10702768; doi:10.3389/fmolb.2023.1327893)
Supplement: Supplementary file 1 [file DataSheet1.ZIP › Supplementary Materials/MR plots for tongue/tongue═╝/Colorectal cancer/pheno.982_to_colorectal cancer_leave_one_out.pdf]

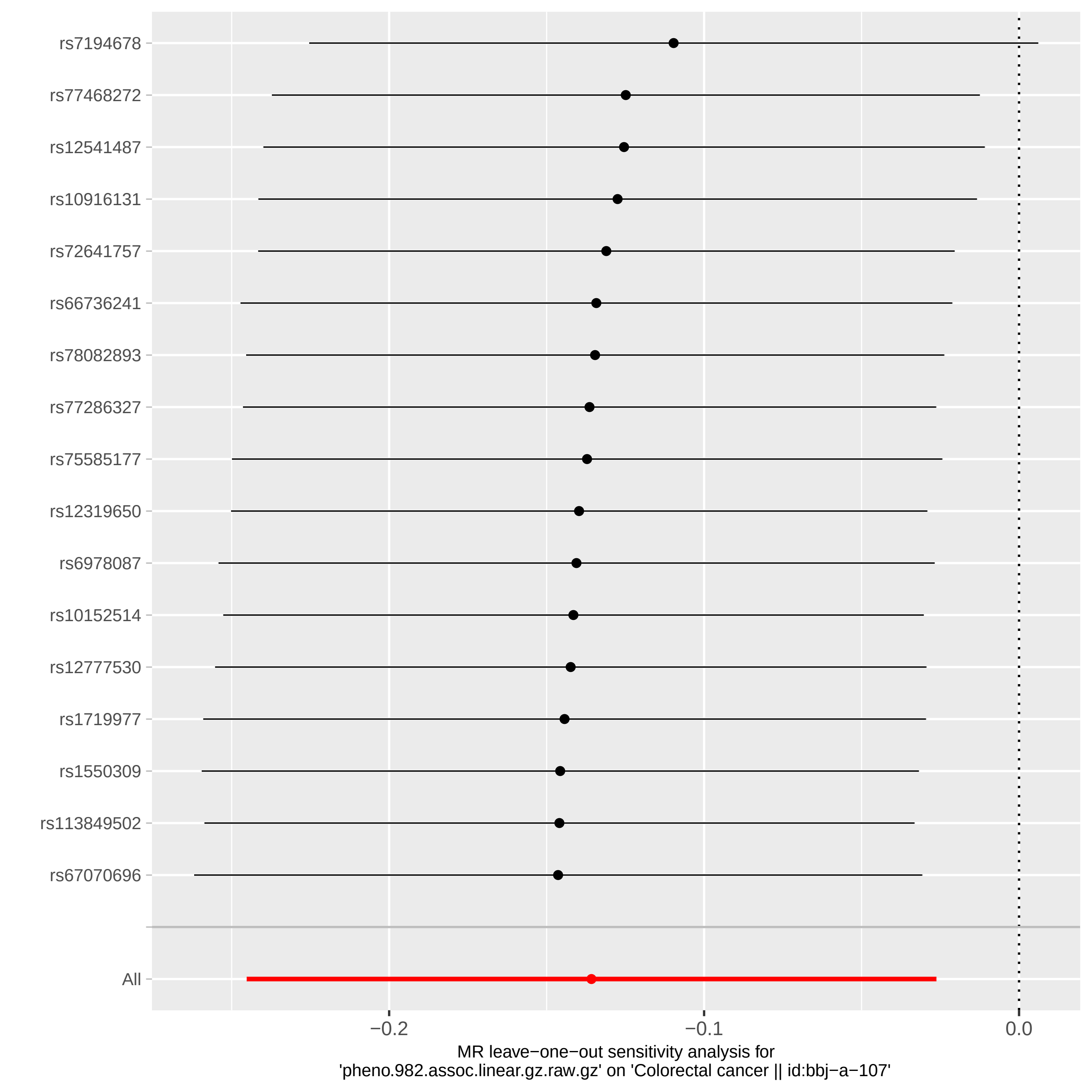
rs7194678
rs77468272
rs12541487
rs10916131
rs72641757
rs66736241
rs78082893
rs77286327
rs75585177
rs12319650
rs6978087
rs10152514
rs12777530
rs1719977
rs1550309
rs113849502
rs67070696
All
−0.2
−0.1
0.0
MR leave−one−out sensitivity analysis for
'pheno.982.assoc.linear.gz.raw.gz' on 'Colorectal cancer || id:bbj−a−107'
